# Supplementary material for: How Central Is the Domestic Pig in the Epidemiological Cycle of Japanese Encephalitis Virus? A Review of Scientific Evidence and Implications for Disease Control
Source: Viruses. 2019 Oct 15;11(10):949. doi: 10.3390/v11100949 (PMC6832429; doi:10.3390/v11100949)
Supplement: Supplementary file 1 [file viruses-11-00949-s001.pdf]

Supplementary Material

SM 1

Variability of the JEV diagnostic tests for pigs

Diagnostic tests for JEV in pigs are essential both in experimental studies and in order to identify regions or period being at risk and epidemiological processes. Indeed, clinical signs in pigs are complex and unspecific. However, the main difficulty to assess the exposition of pigs to JEV lies in the variability of the tests available and used, and the cross reactivity and protection within and across the different flavivirus serocomplexes (1–5). As a necessary preliminary, we here briefly present the different tests used for JEV detection in swine.

Virus detection and isolation and genome detection

Virus isolation, which is the definitive diagnosis, is possible through different technics. Some old studies used suckling mice intracerebral inoculation (SMIC) of samples of tissues to be tested. Mice were intracranially inoculated and monitored in order to detect neurological symptoms or death in a defined time laps. Virus isolation is now made by infection of cells: mammalian cells (Vero cells) or mosquito cells (*Aedes albopictus* C6/36 cells). JEV is then either confirmed by observation of cytopathic effect on cells. Reverse transcriptase polymerase chain reaction (RT-PCR) is the reference technique to detect the virus genome, being extremely sensitive and specific (6–9). Compared to cell culture and virus isolation attempt, RT-PCR remains more sensitive (10).

Serological assays

The plaque reduction neutralization test (PRNT) is the standard technique for the serological diagnosis of flavivirus infections. The test relies on the protection of cells when neutralizing antibodies are present in the serum and able to neutralize a known quantity of virus. Ability of neutralizing antibodies to reduce the number of lysis plaques in a monolayer cell culture is visually quantified. A sample is considered positive if a certain plaque reduction threshold compared to the control serum is reached. The standard protocol provides for a threshold of 90% reduction of lysis plaques ($PRNT_{90}$). However, several studies use a lower threshold of 80% or even 50%, in order to increase sensibility of the test by accepting a reduction of its specificity (11,12). Enzyme-Linked Immunosorbent Assays are based on a colorimetric reaction for which the color intensity is related to the antibody concentration. Various ELISA kits are available (« porcine JE-IgG, SunRed; ID screen WN ID VET). It is an interesting tool for serological survey as a first screening (11,12). Although ELISA is used to be carried out on serum, flavivirus antibodies can also be detected by ELISA on meat juice, supposed to make surveillance in wild animals easier (13). The haemagglutination inhibition assay (HIA) is used in some laboratories, especially for bird surveys. The HIA exploits the ability of viral envelope protein to aggregate erythrocytes in the absence of neutralizing envelope antibodies (14). HIA and ELISA are both subject to cross-reactivity in the JEV serocomplex and with other flavivirus serocomplexes. Positive samples should then be confirmed with SNT if one wants to conclude on specificity JEV seroprevalence (11,15).

References

1. Calisher CH, Karabatsos N, Dalrymple JM, Shope RE, Porterfield JS, Westaway EG, et al. Antigenic relationships between flaviviruses as determined by cross-neutralization tests with polyclonal antisera. J Gen Virol. 1989 Jan;70 (Pt 1):37–43.

2. Mansfield KL, Horton DL, Johnson N, Li L, Barrett ADT, Smith DJ, et al. Flavivirus-induced antibody cross-reactivity. Journal of General Virology. 2011;92(12):2821–9.

3. Holbrook MR, Shope RE, Barrett ADT. Use of Recombinant E Protein Domain III-Based Enzyme-Linked Immunosorbent Assays for Differentiation of Tick-Borne Encephalitis Serocomplex Flaviviruses from Mosquito-Borne Flaviviruses. Journal of Clinical Microbiology. 2004 Sep 1;42(9):4101–10.

4. Goverdhan MK, Kulkarni AB, Gupta AK, Tupe CD, Rodrigues JJ. Two-way cross-protection between West Nile and Japanese encephalitis viruses in bonnet macaques. Acta Virol. 1992 May;36(3):277–83.

5. Nemeth NM, Bosco-Lauth AM, Bowen RA. Cross-protection between West Nile and Japanese encephalitis viruses in red-winged blackbirds (Agelaius phoeniceus). Avian Dis. 2009 Sep;53(3):421–5.

6. Yang DK, Kweon CH, Kim BH, Lim SI, Kim SH, Kwon JH, et al. TaqMan reverse transcription polymerase chain reaction for the detection of Japanese encephalitis virus. Journal of veterinary science (Suwon-si, Korea). 2004;5(4):345–51.

7. Ogawa H, Taira O, Hirai T, Takeuchi H, Nagao A, Ishikawa Y, et al. Multiplex PCR and multiplex RT-PCR for inclusive detection of major swine DNA and RNA viruses in pigs with multiple infections. Journal of Virological Methods. 2009;160(1–2):210–4.

8. Patel P, Landt O, Kaiser M, Faye O, Koppe T, Lass U, et al. Development of one-step quantitative reverse transcription PCR for the rapid detection of flaviviruses. Virol J. 2013 Feb 14;10:58.

9. Eldadah ZA, Asher DM, Godec MS, Pomeroy KL, Goldfarb LG, Feinstone SM, et al. Detection of flaviviruses by reverse-transcriptase polymerase chain reaction. J Med Virol. 1991 Apr;33(4):260–7.

10. Hierholzer JC, Killington RA. 2 - Virus isolation and quantitation. In: Mahy BW, Kangro HO, editors. Virology Methods Manual. London: Academic Press; 1996. p. 25–46.

11. Beck C, Lowenski S, Durand B, Bahuon C, Zientara S, Lecollinet S. Improved reliability of serological tools for the diagnosis of West Nile fever in horses within Europe. PLoS Negl Trop Dis. 2017 Sep 15;11(9).

12. Chernesky MA. 6 - Traditional serological tests. In: Mahy BW, Kangro HO, editors. Virology Methods Manual. London: Academic Press; 1996. p. 107–22.

13. Yonemitsu K, Minami S, Noguchi K, Kuwata R, Shimoda H, Maeda K. Detection of anti-viral antibodies from meat juice of wild boars. The Journal of veterinary medical science. 2019;81(1):155–9.

14. Clarke DH, Casals J. Techniques for hemagglutination and hemagglutination-inhibition with arthropod-borne viruses. Am J Trop Med Hyg. 1958 Sep;7(5):561–73.

15. Maeki T, Tajima S, Ikeda M, Kato F, Taniguchi S, Nakayama E, et al. Analysis of cross-reactivity between flaviviruses with sera of patients with Japanese encephalitis showed the importance of neutralization tests for the diagnosis of Japanese encephalitis. Journal of Infection and Chemotherapy. 2019 May 16
